# Effect of Menopausal Status on Carotid Intima-Media Thickness and Presence of Carotid Plaque in Chinese Women Generation Population

**DOI:** 10.1038/srep08076

**Published:** 2015-01-28

**Authors:** Yong Zhou, Dandan Wang, Xin Yang, Anxin Wang, Xiang Gao, Yuming Guo, Shouling Wu, Xingquan Zhao

**Affiliations:** 1 Department of Neurology, Beijing Tiantan Hospital, Capital Medical University, Beijing, China; 2 China National Clinical Research Center for Neurological Diseases, Beijing, China; 3 Center of Stroke, Beijing Institute for Brain Disorders; 4 Beijing Key Laboratory of Translational Medicine for Cerebrovascular Disease; 5 Department of General Practice, School of General Practice and Continuing Education, Capital Medical University, Beijing, China; 6 Channing Laboratory, Department of Medicine, Brigham and Women's Hospital, and Harvard Medical School, Boston, MA, USA; 7 Department of Nutrition, Harvard University School of Public Health, Boston, MA, USA; 8 Division of Epidemiology and Biostatistics, School of Population Health, University of Queensland, Brisbane, Australia; 9 Department of Cardiology, Kailuan Hospital, Tangshan, China

## Abstract

Menopause is an important physiological stage in women's life. The potential association of menopause with carotid intima-media thickness as well as with occurrence and stability of carotid plaque in Chinese female population is unclear. We conducted a population-based, cross-sectional study by recruiting 2,131 participants aged above 40 years from northeast of China. Carotid intima-media thickness (CIMT), presence of carotid plaque and its stability were evaluated by carotid duplex sonography. Among the participants, 1,133 (53.2%) were identified to be postmenopausal. After adjusting for potential confounding factors, presence of CIMT at 50^th-^ 75^th^ and ≥75^th^ percentiles, carotid plaque and its unstable status were found to be significantly associated with the postmenopausal status (*P* < 0.001). When matched the participants by age, post-menopausal status was still associated with a higher risk of having unstable plaque. Moreover, our data show that postmenopausal status is a risk factor for intracranial arterial stenosis when compared with premenopausal status in the univariate analysis (OR = 1.314, P = 0.043), and such relationship is lost when the confounding factors are adjusted (OR = 0.828, P = 0.225). In conclusion, the vascular risk factors increase as the menopausal status changes. Compared with premenopausal status, postmenopausal status is associated with higher morbidity of CIMT, carotid plaque and its unstable status.

## Background

Menopause is one of the physiological stages in women's life, during which women experience a series of physiological and psychological changes because of some complex processes such as disorder of endocrine system and/or dysregulation of neurological function. These changes, if not treated appropriately, may not only affect women's health, but also may pose the life and work quality of the whole family at risk. Previous studies have indicated that there is significant difference in development of coronary artery disease (CAD) between premenopausal women and age-matched men, and such difference is not obvious between postmenopausal women and age-matched men^1^,^2^,^3^. In addition, estrogens, a group of important female hormones, have vasodilatation and anti-inflammatory functions. It has been shown that endogenous estrogens may play a protective role in the prevalence of CAD irrespective of sex^4^,^5^,^6^,^7^. Howard Hodis and his colleagues found out that in healthy postmenopausal women, using estrogen and sex hormone-binding globulin could reduce subclinical atherosclerosis progression^8^,^9^. The mechanisms may involve that the hormones affect lipid situation, coagulation and fibrinolysis factors and may play important roles in multiple physiological systems, including the adipose/metabolic system, the cardiovascular system and the central nervous system^4^,^10^,^11^,^12^.

Cerebrovascular diseases, including arterial stenosis and arterial plaque formation, are mainly caused by arterial endothelial damage, leading to inflammation and fatty deposits. The gradual damage induces atherosclerosis, reduces the diameter of blood vessels, and then causes vascular stenosis^13^. Kablak and his colleagues reported that carotid intima-media thickness (CIMT) is a strong CAD predictor in both pre- and postmenopausal women, in contrast to that in the menopausal women^14^. While the association of menopause with CIMT and stroke has been suggested in the studies mentioned above, it is still unclear whether different menopause stages can affect CIMT differentially, thereby leading to potential differences in development and stability of carotid plaques in Chinese women population. Here we conducted this population-based study to explore potential factors that can affect CIMT and presence and stability of carotid plaques in different menopause stages, and to assess whether the arterial stenosis differs in their relationships with different menopause stages.

## Results

Out of the studied 2131 female subjects, 998(46.8%) patients were premenopausal and 1133(53.2%) were postmenopausal. When comparing premenopausal and postmenopausal groups, parameters such as age, BMI, prevalence of hypertension, diabetes mellitus and dyslipidemia and blood concentrations of uric acid, C-reactive protein and homocysteine were found to be significantly different. Prevalence of intracranial arterial stenosis (ICAS) was also significantly lower in premenopausal group than in postmenopausal group (Table 1).

In chi-squared test, we compared the menopausal status according to CIMT, carotid plaque and its stability. In the carotid plaque group, 229(22.9%) were premenopausal, and 588(51.9%) were postmenopausal, and the difference is statistically significant (P < 0.001). In the carotid plaque group with stability as a factor, 64(27.9%) with unstable carotid plaque were premenopausal, and 290(49.3%) with unstable carotid plaque were postmenopausal (P < 0.001). When CIMT was compared between different groups of menopausal status, the median of CIMT was 0.70 mm in the premenopausal group and 0.80 mm in postmenopausal group (P < 0.001) (Table 2).

In univariate analysis, postmenopausal status was found to be associated with the higher presence of carotid plaque (OR: 3.623, 95%CI: 3.002, 4.372; P < 0.001) and unstable carotid plaque rate (OR: 2.509, 95%CI: 1.802, 3.493; P < 0.001). With regard to CIMT, compared with CIMT ≤25^th^ percentile, CIMT with 50^th^–75^th^and ≥75^th^ percentiles were significantly higher in postmenopausal group than premenopausal group (OR: 2.473, 95%CI: 1,934, 3.162; P < 0.001 and OR: 7.964, 95%CI: 6.008, 10.556; P < 0.001 respectively).No significant difference was found between the CIMT ≤25^th^ percentile and the 25^th^–50^th^ percentile groups (OR: 1.037, 95%CI: 0.818, 1.315; P = 0.764) (Table 3).

Since menopausal status is strongly related to age, age was not adjusted when we first examined the potential association between the menopausal status and carotid intima-media thickness and presence of carotid plaque. In the multivariate analysis, the menopausal status and other factors were included as dependent or independent variables. After adjusting for all those parameters including body mass index (BMI), presence of hypertension, diabetes mellitus and dyslipidemia, and blood concentration of uric acid, C-reactive protein and homocysteine, the menopausal status was still found to be significantly associated with CIMT (P < 0.001).In order to exclude the interference of age to our results, 223 post-menopausal women were successfully matched to another 223 pre-menopausal women based on their age. The mean age of these 223 pairs was 49 years (SD: 3.7 years), ranging from 40 years to 63 years. We found that the menopausal status was still associated with unstable plaque even after adjusting other related factors (1.766 (0.849, 3.674)) (Table 3).

We also found that postmenopausal status is a risk factor of ICAS when compared with premenopausal status in the univariate analysis (1.314 (1.009, 1.710), p < 0.05), and such relationship became non-significant when other related parameters were adjusted (0.828 (0.610, 1.123), p = 0.225) (Table 4).

## Discussion

We here show in this community-based study with relatively large subject size that menopausal status is significantly associated with CIMT as well as the development and stability of carotid plaques in a Chinese women population. After adjusting for other risk factors (including BMI, presence of hypertension, diabetes mellitus and dyslipidemia and blood concentration of uric acid, C-reactive protein and homocysteine), we further demonstrate that occurrence of CIMT and formation of carotid plaques, particularly the unstable ones, were also significantly higher in postmenopausal women compared with premenopausal women. Since menopausal status is strongly related to age, we could not separate age and menopausal status apart and only analysis the effect of menopausal status to CIMT and plaque. In age- matched group, although no significant results came out in the association between menopausal status and CIMT, carotid plaque and its stability, we still found that there is a trend that post-menopausal status was associated with a higher risk of having unstable plaque (instead of stable plaque) than pre-menopausal status even after we fully controlled for age through exact matching. So we think our results still tells a trend between women's menopausal status and carotid condition.

Increased CIMT and presence of unstable carotid plaques are commonly seen in clinical patients and are all closely related to development of ischemic stroke. It has been shown that increased CIMT is a predictive marker for onset of atherosclerosis and is associated with the vascular events^15^. Similar to the previous studies^16^,^17^, we also used mean CIMT for assessing the left and right CCAs together. Formation of carotid plaques has been regarded as a marker for the advanced arterial injury and early stages of atherosclerosis^17^,^18^. Other than CIMT and carotid plaques, ICAS is also a known risk factor of stroke. Studies have shown that distribution of ICAS varies between different ethnic groups^19^. In contrast to extracranial atherosclerosis and arterial stenosis, intracranial atherosclerosis and arterial stenosis are more common in Asian, African, Spanish and Portuguese populations^20^. ICAS-related stroke comprises 33% to37% of all ischemic strokes in Chinese population^21^,^22^. In this study, we show that the prevalence of CIMT, carotid plaque and its unstable status as well as ICAS are all higher in the postmenopausal women as compared with the premenopausal women. With respect to potential biological mechanisms, menopause in females is mainly caused by changes in levels of certain sex hormones, including estradiol and testosterone^23^. Sex hormone-binding globulin (SHBG) is a bridging protein which binds to testosterone and estradiol and transfers them to the target organs. When the level of sex hormones decreases, SHBG binds preferentially to testosterone^24^. When women reach their perimenopausal and postmenopausal life stages, the level of SHBG and estradiol decreases, and the follicle stimulating hormone (FSH) increases, but the testosterone and free androgen index increases at first and then decreases on her postmenopausal status^25^. All these changes might contribute to the development of CIMT and carotid plaques.

Using CIMT, carotid plaque and its status of stability as the markers of atherosclerosis, our study confirms the results of previous investigations that menopause transition in women is associated with accelerated subclinical atherosclerosis progression^26^,^27^,^28^. However, the potential association of the status of female menopause with the formation and stability status of carotid plaques and ICAS has not yet been examined in those studies, particularly for Chinese women population. Therefore, our results presented in this study further extends the findings of previous investigations by including not only CIMT, but also carotid plaque and its stability as well as ICAS as testing factors that demonstrate associations with menopause. Since estrogen plays a protective role in cardiovascular diseases (CVD), when women reach their menopausal life stages, their levels of estrogen drop rapidly, and therefore risk for developing the subclinical atherosclerosis can significantly increase in these population. Due to readiness of measuring CIMT, carotid plaque and its stability status, utilization of these markers for assessing the subclinical atherosclerosis may be useful in predicting the morbidity of stroke.

A few limitations of the current study are acknowledged. First, the study is based on a randomly selected subgroup of population from the larger reference Kailuan Study where the employees and retirees of the Kailuan Company are included. One may argue that despite its large size of subjects, the Kailuan Study population may not be representative for the general population of the City of Tangshan or other parts of China. In addition, the study population was selected using a stratified random sampling method by age based on the data from the 2010 Chinese National Census. Second, this study is a cohort study, we did not match the participants by age at its baseline, so there might be a bias to our results. Thus, few participants in our study took estrogen at their postmenopausal status, and we did not investigate participants' diet habit which could influence participants' estrogen level, like whether they take high soy diet or not, so further studies are needed to investigate the effect of estrogen in postmenopausal women's health when compared with age- matched premenopausal women, especially in Chinese women.

In conclusion, after adjusting for potential risk factors, our analyses in this study show that the female postmenopausal status is significantly associated with modest and severe increase in the thickness of CIMT (CIMT at 50^th-^ 75^th^ and ≥75^th^ percentiles) as well as with the formation and stability of carotid plaques in Chinese women population. When matched the participants by age, post-menopausal status was still associated with a higher risk of having unstable plaque. In contrast, the association between the postmenopausal status and mild increase in CIMT thickness (25^th^–50^th^ percentile) or ICAS is less significant.

## Methods

### Study participants

The Asymptomatic Polyvascular Abnormalities Community study (APAC) is a community-based observational study aiming to investigate the epidemiology of asymptomatic polyvascular abnormalities in Chinese adult populations^29^,^30^. The study cohort is derived from a previously described reference population of the Kailuan study that includes a total of 101,510 adult human subjects, mainly the employees and retirees of the Kailuan (Group) Co. Ltd, one of the largest coal mining industrial companies located in City of Tangshan, Hebei Province in China. The Tangshan city has approximately 7.2 million people as of 2006 and is situated about 150 km away from southeast of Beijing. Using an age and gender stratified random sampling method based on the information from the 2010 Chinese National Census, a subject size of 7000 participants more than 40 years old was randomly selected from the Kailuan cohort between June 2010 and June 2011. A total of 5,852 subjects agreed to participate in the APAC study and 5,816 people were eventually completed for baseline data collection. Among these 5,816 individuals, 376 subjects did not meet the inclusion criteria which were detailed as following: (1) no history of stroke, transient ischemic attack, and coronary disease at baseline as assessed by a validated questionnaire; and (2) absence of neurologic deficits indicating previous stroke as examined by experienced physicians. A total of 5440 participants were eligible and enrolled in the APAC study where 2183 participants are women. 52 Subjects with incomplete information regarding menopause stages, CIMT, presence of carotid plaque and its stability were further excluded. Finally, a total of 2131 participants were eligible and included in this study.

The study was approved by the Ethics Committees of the Kailuan General Hospital and the Beijing Tiantan Hospital. All experiments in this study were performed according to the guidelines from the Helsinki Declaration and written informed consent was obtained from all participants. Subjects were also informed of abnormal findings and recommended treatment.

Structured interviews with standardized questionnaire were performed by trained investigators. The questionnaire was designed to obtain information on the demographic and socioeconomic background, level of education, self-reported income, menopause status, age of pausimenia and history of major medical disorders such as hypertension, diabetes and dyslipidemia, alcohol consumption and smoking. Anthropometric indices included height and weight. Body mass index (BMI) was calculated as body weight (kg) divided by the square of body height (m2). Smoking was defined as at least one cigarette per day for more than a year. Alcohol consumption was defined as intake of at least 100 ml of liquor per day for more than one year. Smoking or drinking cessation was considered only if it lasted for at least one year.

### Assessment of Carotid Plaque

**Figure 1:**
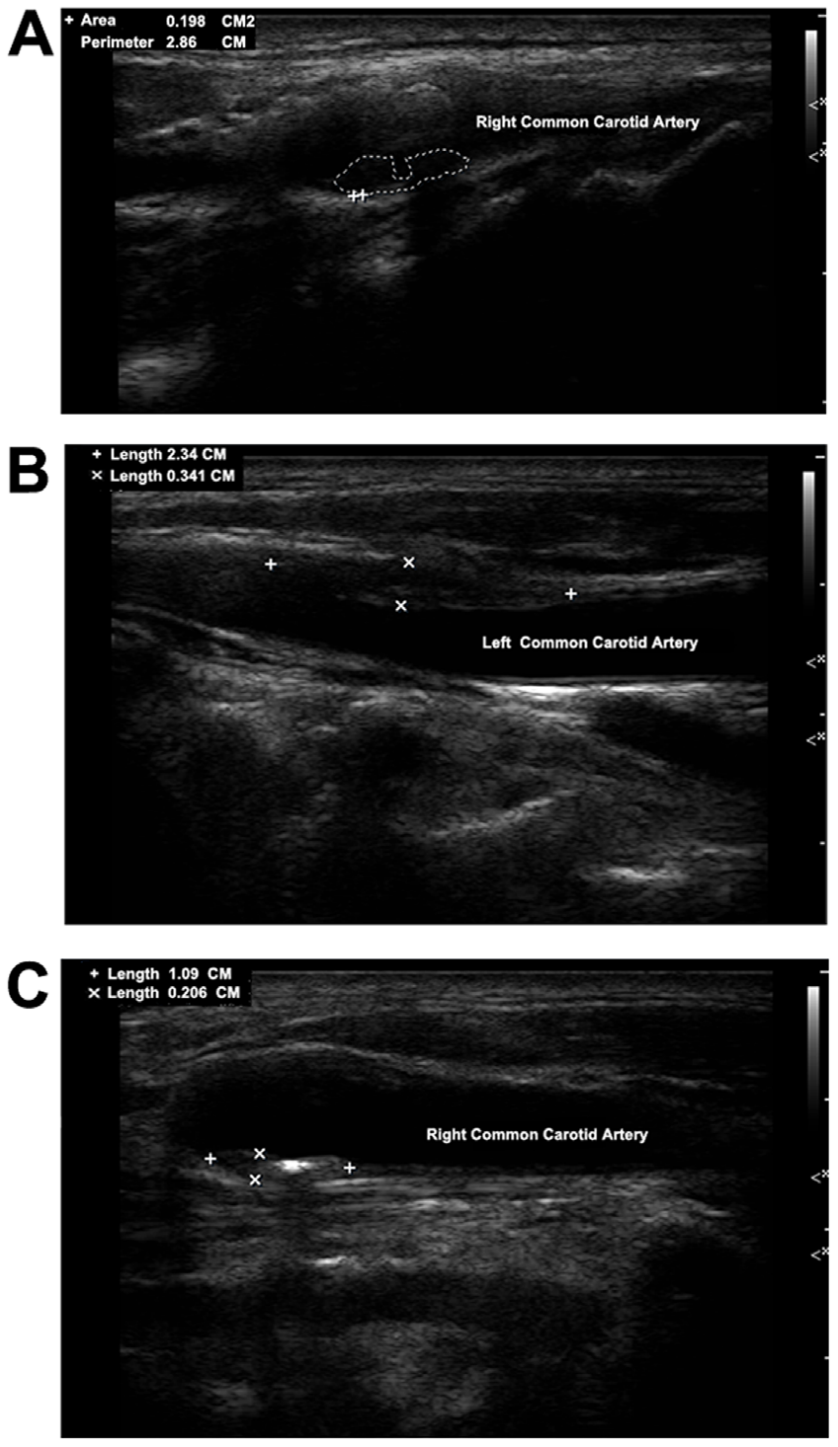
Representative ultrasound images for different types of advanced carotid plaques in study participants; (A). ulcerated plaque; (B). plaque with low-level echo; (C). plaque with heterogeneous echo.

Carotid plaques were evaluated for plaque complexity and advancement by certified sonographers using ultrasounds (Philips iU-22 ultrasound system, Philips Medical Systems, Bothell, WA). Bilateral carotid arteries including common carotid arteries, carotid bifurcation, internal carotid artery and external carotid artery were all examined for study participants in a supine position, head turning to the contralateral side. Both sides of carotid arteries were extensively evaluated. CIMT was measured at the far wall of the common carotid artery (CCA) proximal to the bifurcation, along a plaque-free segment of ≥10 mm long at each side, with a quality index of ≥0.60. CIMTs of bilateral CCAs were then averaged to get a mean CIMT value for each subject. CIMTs were measured twice by the same technician for subsequent assessment of intrarater reproducibility^16^. Carotid plaque was defined as a focal structure either encroaching into the arterial lumen of at least 0.5 mm or 50% of the surrounding IMT value, or demonstrating as a thickness of 1.5 mm from the intima-lumen interface to the media adventitia interface. The carotid ultrasound examination results were then reviewed by two independent operators. Discrepancies between their evaluations were resolved by consensus. In this study, advanced or complicated carotid plaques were defined based on: (1) plaques with incomplete fibrous cap or ulcerated plaques, according to the plaque morphology, and (2) plaques with low-level or heterogeneous echoes, according to the plaque echodensity. Representative ultrasound images for different types of plaques were provided (Figure 1).

### Assessment of arterial stenosis

Transcranial Doppler sonography was performed by two experienced neurologists using portable devices (EME Companion, Nicolet, Madison, WI, USA). ICAS diagnosis was made according to the peak flow velocity criteria that was published and validated against MR angiography and clinical outcomes^31^,^32^. Briefly, occlusive arteries were defined by a peak systolic flow velocity of >140 cm per second for the middle cerebral artery, >120 cm per second for the anterior cerebral artery, >100 cm per second for the posterior cerebral artery and vertebra-basilar artery, and >120 cm per second for the siphon internal carotid artery. In addition, information regarding age of the patients, presence of disturbance in the echo frequency, turbulence, and whether the abnormal velocity was segmental was also taken into consideration for the diagnosis of ICAS. Subjects without a good temporal window were considered having no stenosis. Patients were classified as having an occlusive disease if at least one of the studied arteries showed evidence of stenosis or occlusion.

For assessment of an extracranial arterial stenosis (ECAS), each participant underwent a bilateral carotid duplex sonography. ECAS was defined by a peak systolic blood flow velocity ≥125 cm/s and vertical artery peak systolic blood flow velocity of ≥170 cm/s in the common carotid artery or internal carotid artery. A carotid artery stenosis (≥50%) was graded based on recommendations from the Society of Radiologists in Ultrasound Consensus Conference^33^.

The ankle brachial index (ABI) was calculated using a standard method^34^. Systolic blood pressure was measured with a handheld 5-MHz Bidirectional Doppler probe (Hokanson MD6 Doppler with MD6VR Chart Recorder; Bellevue, Wash). Pressures in each leg were determined and the ABI was calculated separately for each leg. An ABI <0.90 in either leg was considered as a marker for the presence of a peripheral arterial disease (PAD), and an ABI ≥0.90 was considered normal. Elevated ABI values of ≥1.40 suggested poorly compressible leg arteries and were excluded from the statistical analysis.

Blood samples were collected from the antecubital vein in the morning after an overnight fasting and transfused into vacuum tubes containing EDTA. The blood samples underwent a biochemical analysis for measurement of the blood concentrations of uric acid, c-reaction protein and homocysteine.

### Statistical analysis

Statistical tests were performed by a commercially available software program (SPSS software, version 21.0, IBM-SPSS, Chicago, USA). Depending on the distribution of the parameters, their means were compared using Student's t-test or the non-parametric Wilcoxon-test and Mann-Whitney-test. The chi-squared test was used for comparison of categorical variables. Binary logistic regression models were applied to assess the associations between the presence of arterial stenosis and menopause stages with other parameters such as age and sex adjusted. Odds ratios (ORs) were calculated and their 95% confidence intervals (CI) were presented. The null hypothesis was rejected for P < 0.05.

## Author Contributions

D.W. and Y.Z. analyzed, interpreted the data and drafted the manuscript. X.Z. and S.W. conceived and designed the research. X.Z. and X.G. handled funding and supervision. A.W. acquired the data. Y.G. made critical revision of the manuscript. X.Y. prepared figure 1. Y.Z. and D.W. share the first authorship.

## Figures and Tables

**Table 1:** Baseline Characteristics of study participants and their Univariate Association with menopausal status

	premenopause	postmenopause	P value
No.	998	1133	
Age, y	44.7 (42.4, 46.8)	56.0 (52.5, 62.8)	<0.001
BMI, kg/m^2^	24.2 (22.0, 26.2)	24.7 (22.6, 27.2)	<0.001
Smoking, %	13 (1.3)	22 (1.9)	0.306
Drinking, %	5 (0.5)	1 (0.1)	0.105
Hypertension, %	196 (19.6)	577 (50.9)	<0.001
Diabetes, %	44 (4.4)	168 (14.8)	<0.001
Dyslipidemia, %	321 (32.2)	655 (57.8)	<0.001
Uric acid, umol/l	229.0 (192.3,271.0)	261.0 (215.0, 315.6)	<0.001
CRP, mg/l	0.8 (0.4, 1.5)	1.2 (0.7, 2.6)	<0.001
HCY, umol/l	9.2 (6.3, 12.5)	11.7 (8.9, 15.7)	<0.001
ICAS	106 (10.6)	153 (13.5)	0.046
ECAS	31 (3.1)	287(2.4)	0.351
PAD	22 (2.3)	33 (3.0)	0.340

Data are median (25% interquartile range, 75% interquartile range) or n (%).

Arterial Stenosis: Intracranial Artery Stenosis (ICAS) or Extracranial Artery Stenosis(ECAS) or Peripheral Artery Disease(PAD); BMI: Body Mass Index; CRP: C Reaction Protein; HCY: Homocysteine.

Premenopausal group: age more than 40 years with regular bleeding without hormone replacement therapy;

Postmenopausal group: with an amenorrhea; or with regular bleeding with hormone replacement therapy.

**Table 2:** Analysis of carotid intima-media thickness (CIMT) and presence of carotid plaque and its stability in different menopausal groups

	premenopause	postmenopause	P value
Carotid Plaque	229 (22.9)	588 (51.9)	<0.001
Carotid Plaque Unstable	64 (27.9)	290 (49.3)	<0.001
Carotid Intima-Media Thickness	0.70 (0.60, 0.75)	0.80 (0.70, 0.90)	<0.001

Data are median (25% interquartile range, 75% interquartile range) or n (%).

**Table 3:** Crude and Adjusted Odds Ratios (ORs) and 95% CI of Postmenopausal status for carotid plague, stability and CIMT compared to premenopausal status

		Crude		Adjusted	
	No. of events/all	OR (95%CI)	P value	OR (95%CI)	P value
Carotid Plaque	588/817	3.623 (3.002, 4.372)	<0.001	2.335 (1.891, 2.884)	<0.001
Age matched		0.922 (0.622, 1.368)	0.688	0.896 (0.591, 1.358)	0.604
Carotid Plaque Unstable	290/354	2.509 (1.802, 3.493)	<0.001	2.182 (1.506, 3.161)	<0.001
Age matched		1.909 (0.945, 3.856)	0.071	1.766 (0.849, 3.674)	0.128
CIMT in quartiles					
Quartile 1 (< = 0.65 mm)	236/630	1.000		1.000	
Quartile 2 (0.66–0.70 mm)	205/535	1.037 (0.818, 1.315)	0.764	0.964 (0.744, 1.249)	0.781
Age matched		0.760 (.0479, 1.203)	0.241	0.742 (0.461, 1.196)	0.220
Quartile 3 (0.71–0.80 mm)	277/464	2.473 (1.934, 3.162)	<0.001	1.716 (1.292, 2.281)	<0.001
Age matched		1.223 (0.714, 2.093)	0.464	1.207 (0.683, 2.131)	0.517
Quartile 4 (>0.80 mm)	415/502	7.964 (6.008, 10.556)	<0.001	4.259 (3.068, 5.912)	<0.001
Age matched		1.057 (0.602, 1.856)	0.846	0.905 (0.485, 1.692)	0.755

CIMT: Carotid Intima-Media Thickness.

Adjusted by BMI, hypertension, diabetes, dyslipidemia, C-reaction protein, uric acid and homocysteine.

**Table 4:** Crude and Adjusted Odds Ratios (ORs) and 95% CI of postmenopausal status for ICAS

		Crude		Adjusted for other risk factors	
	No. of events/all	OR (95%CI)	P value	OR (95%CI)	P value
ICAS	588/817	1.314 (1.009, 1.710)	0.043	0.828 (0.610, 1.123)	0.225

Adjusted by BMI, hypertension, diabetes, dyslipidemia, C-reaction protein, uric acid and homocysteine.
